# The Promise of Epigenetics Research in the Treatment of Appendiceal Neoplasms

**DOI:** 10.3390/cells12151962

**Published:** 2023-07-29

**Authors:** Luisa Ladel, Wan Ying Tan, Thanushiya Jeyakanthan, Bethsebie Sailo, Anup Sharma, Nita Ahuja

**Affiliations:** 1 Surgical Oncology Research Laboratories, Division of Surgical Oncology, Department of Surgery, Yale School of Medicine, Yale University, New Haven, CT 06519, USA; luisa.ladel@nuvancehealth.org (L.L.); wanying.tan@nuvancehealth.org (W.Y.T.); thanushiya.jeyakanthan@nuvancehealth.org (T.J.); bethsebie.sailo@yale.edu (B.S.); anup.sharma@yale.edu (A.S.); 2 Affiliated Internal Medicine Residency Program at Norwalk Hospital, Department of Internal Medicine, Norwalk Hospital, Yale University, Norwalk, CT 06850, USA; 3 Department of Pathology, Yale School of Medicine, New Haven, CT 06519, USA

**Keywords:** appendiceal cancer, epigenetics, translational research, cancer biomarkers, gastrointestinal cancer, precision oncology, epigenetic-specific biomarker

## Abstract

Appendiceal cancers (AC) are a rare and heterogeneous group of malignancies. Historically, appendiceal neoplasms have been grouped with colorectal cancers (CRC), and treatment strategies have been modeled after CRC management guidelines due to their structural similarities and anatomical proximity. However, the two have marked differences in biological behavior and treatment response, and evidence suggests significant discrepancies in their respective genetic profiles. In addition, while the WHO classification for appendiceal cancers is currently based on traditional histopathological criteria, studies have demonstrated that histomorphology does not correlate with survival or treatment response in AC. Due to their rarity, appendiceal cancers have not been studied as extensively as other gastrointestinal cancers. However, their incidence has been increasing steadily over the past decade, making it crucial to identify new and more effective strategies for detection and treatment. Recent efforts to map and understand the molecular landscape of appendiceal cancers have unearthed a wealth of information that has made it evident that appendiceal cancers possess a unique molecular profile, distinct from other gastrointestinal cancers. This review focuses on the epigenetic landscape of epithelial appendiceal cancers and aims to provide a comprehensive overview of the current state of knowledge of epigenetic changes across different appendiceal cancer subtypes, highlighting the challenges as well as the promise of employing epigenetics in the quest for the detection of biomarkers, therapeutic targets, surveillance markers, and predictors of treatment response and survival in epithelial appendiceal neoplasms.

## 1. Introduction

Although appendiceal cancers (AC) are rare, there has been a trend of increasing incidence of appendiceal malignancies since 2000, based on the National Cancer Database (NCDB). A 54% increase in appendiceal neoplasms in the USA over the past 10-year period has been noted, with a reported approximate incidence of 0.12 to 2.6 cases per million people per year, in line with incidence reports from other North American and European countries [1,2,3,4,5,6]. Unfortunately, no incidence report is available for 2022, and no data regarding estimated global incidence exist. Epidemiological studies on appendiceal neoplasms from European and North American countries do not show any significant sex-based difference in incidence for most appendiceal cancer subtypes, except for appendiceal adenocarcinomas, which are more common in men, and neuroendocrine tumors, which are slightly more common in females [7,8]. Neuroendocrine appendiceal tumors have been observed to occur more frequently under the age of 50 years, while other appendiceal cancer subtypes appear more frequently with older age [1,2,9,10,11,12]. The 5-year survival rates for neuroendocrine and low-grade neoplasms of the appendix vary around 67–97%. Meanwhile, lower survival rates have been reported for more advanced and malignant histological subtypes, although specific statistics are not available due to the rare nature of these tumors [8]. 

Appendiceal cancers are commonly diagnosed intraoperatively during appendectomies [6,13]. Recent years have seen a shift toward nonoperative management of acute appendicitis [14,15,16]. This paradigm shift may contribute to missed or late diagnosis of appendiceal cancer. Hence, efforts to stratify and identify high-risk individuals and early-stage appendiceal cancers are crucial. 

Genomic subtyping has been previously performed in attempts to predict biological behavior and clinical outcomes of appendiceal cancers. However, findings have remained inconclusive, and therefore, further molecular studies are needed to improve therapeutic strategies and develop molecular biomarkers for screening, early diagnosis, monitoring, and surveillance. In addition, although there is evidence that appendiceal cancers differ from the pathophysiology of other gastrointestinal cancers based on molecular studies, our understanding of the pathophysiology of appendiceal cancer is limited and remains to be further elucidated [17,18,19,20,21]. Cancer epigenetics have been shown in recent years to play a key role in the pathophysiology of gastrointestinal neoplasms, leading to discoveries and the development of biomarkers for detection, monitoring, surveillance, and therapeutic strategies [22]. This review aims to summarize the current understanding of the molecular biology of appendiceal cancer, focusing on molecular epigenetics and its potential clinical applications.

## 2. The Evolution of Appendiceal Cancer Classification

**Figure 1:**
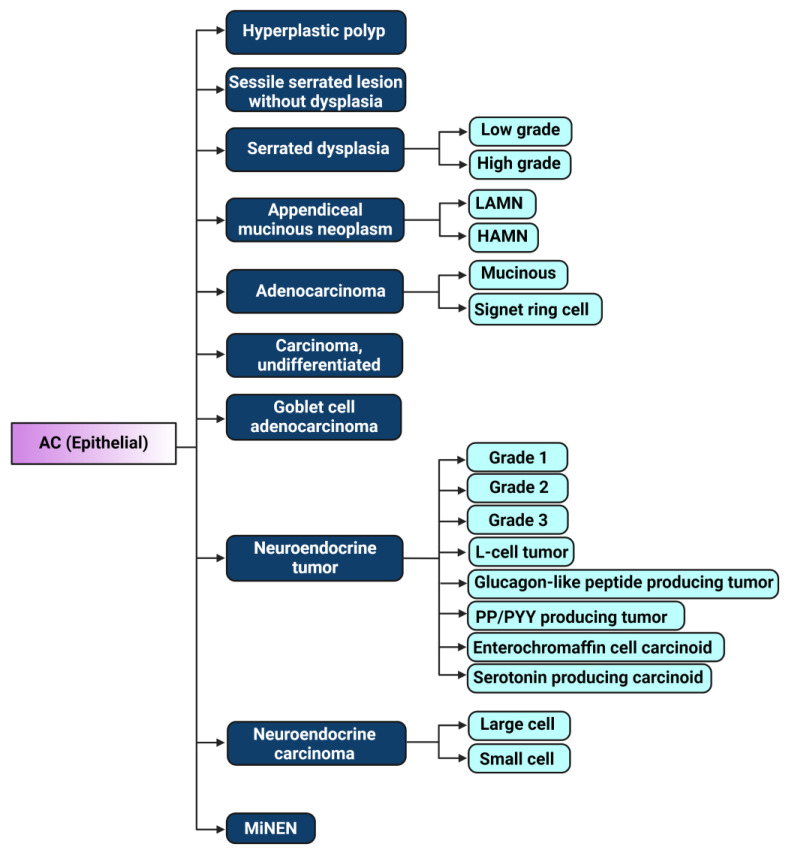
Illustration of 2019 WHO classification of epithelial appendiceal cancers [23]. LAMN: low-grade appendiceal mucinous neoplasm, HAMN: high-grade appendiceal mucinous neoplasm, PP/PYY: pancreatic polypeptide/peptide YY, MiNEN: mixed neuroendocrine–non-neuroendocrine neoplasm. Figure created with biorender.com, accessed on 19 July 2023.

The classification of appendiceal cancers has been a dynamic subject, with the most recent changes made in the 2019 WHO classification. Although histomorphological classification remains the gold standard, histopathology does not predict survival or treatment response differences except in appendiceal signet ring cell carcinoma and malignant carcinoids. The extent of disease at diagnosis is a more important predictor of survival than histology [6]. According to the 2019 WHO histopathological classification, appendiceal tumors include hyperplastic polyps, sessile serrated lesions without dysplasia, low- and high-grade serrated lesions with dysplasia, low- and high-grade mucinous neoplasm, mucinous adenocarcinoma, signet ring cell adenocarcinoma, undifferentiated adenocarcinoma, goblet cell adenocarcinoma, and various subtypes of neuroendocrine tumor and neuroendocrine carcinomas (small and large cells), as well as mixed neuroendocrine–non-neuroendocrine neoplasms (Figure 1) [23,24]. Non-epithelial tumors of the appendix, such as hematolymphoid or mesenchymal tumors, are not addressed in this review.

A subset of epithelial cells in the gastrointestinal tract, including cells within the appendix, produce a substance called mucin which acts as a protective lining for the intestinal lumen. Malignant transformations of these mucin-producing epithelial cells based on histopathological grading are classified as low-grade appendiceal mucinous neoplasm (LAMN) or high-grade appendiceal mucinous neoplasm (HAMN). Both LAMN and HAMN are histologically diagnosed in the absence of infiltrative growth. As per the consensus statements from Peritoneal Surface Oncology Group International (PSOGI), the term mucinous adenocarcinoma should be reserved only for lesions with infiltrative invasion, which demarcates it from LAMN and HAMN [25]. Primary signet ring cell adenocarcinoma of the appendix, named for its tumor cells resembling a signet ring on microscopy, is an exceedingly rare entity, and little is known of its discrete characteristics [25]. Although some studies did not regard signet cell adenocarcinoma as an individual entity since the presence of signet ring cells is a histologic feature that may or may not be present in both colonic-type and mucinous adenocarcinoma, many studies suggest that it should be considered separately from other mucinous neoplasms, mainly because of its poor prognosis [25,26,27]. This distinction was adopted in the 2019 WHO classification system, and signet ring cell adenocarcinoma is now considered a higher histopathologic tumor grade compared to LAMN and HAMN. Interestingly, although both LAMN and HAMN are inherently noninvasive tumors, rupture of the appendix secondary to mucinous tumor growth can cause pseudomyxoma peritonei (PMP), with the term PMP being a strictly clinical term for apparent mucinous ascites or peritoneal mucin deposition [28]. Three categories of PMP were defined by the PSOGI consensus based on histomorphology, including low grade, high grade, and high grade with signet ring cells, grouping signet ring cell carcinoma with HAMN [25]. 

Goblet-cell-derived tumors also need further studies to further characterize and understand their natural history and prognosis. Appendiceal goblet cells give rise to a mixed tumor, which, while characterized by the presence of intestinal-type goblet cells, also contains neuroendocrine elements, and due to its diverse nature was traditionally described as goblet cell carcinoid. However, recent studies have shown that these tumors are closer to adenocarcinoma than neuroendocrine carcinoma based on both immunohistochemical profile and biological behavior and are rarely associated with hormone hypersecretion syndromes [29]. Goblet cell adenocarcinoma (GCA) is a rare tumor almost exclusively found in the appendix [30]. Its disease course varies, in part depending on grade and depth of invasion. These characteristics have led to changes in nomenclature over time, with GCA previously termed adenocarcinoid, mucinous carcinoid, composite goblet cell carcinoid-adenocarcinoma, adenocarcinoma ex-goblet carcinoid, crypt cell carcinoma, and, more recently, goblet cell carcinoma or goblet cell carcinoid (GCC). The evolving nomenclature has led to difficulties and inconsistencies in diagnosis and reporting, obscuring GCA’s exact incidence and survival rates [29,31]. 

With the improvement of our understanding of the histopathology of appendiceal cancers, the 2019 WHO classification of appendiceal cancers has provided a better distinction between different subtypes of appendiceal cancer. With the emergence of next-generation sequencing (NGS), multiple studies on genomic profiling of appendiceal cancers were performed in an attempt to supplement histopathological classification. A large study with 495 appendiceal tumor samples (53 GCAs, 428 appendiceal adenocarcinomas (AA), and 14 ANETs) aimed to build molecular signatures of appendiceal neoplasms via a 592-gene panel and immunohistochemistry (IHC). Molecular profiling of GCAs compared to appendiceal adenocarcinomas (AA) and ANETs found that GCAs had lower mutation rates in *KRAS*, GNAS, and *APC* than AA. They, in turn, have higher mutation rates in *CDH1*, *CHEK2*, *CDC73*, *ERCC2*, and *FGFR2* compared to ANETs (Table 1) [32]. In addition, GCA was found to be more aggressive than stage-matched ANETs. However, there still appears to be a significant knowledge gap regarding reliable molecular markers of GCA [33]. 

A recent publication by Foote et al. defined a new molecular sub-characterization system for mucinous appendiceal adenocarcinoma. It demonstrated a link to survival and overall clinical outcomes by stratifying samples according to the genetic alteration status of three distinct cancer drivers found commonly in colorectal cancer *KRAS*, *GNAS*, and *TP53* [34]. However, the value of this new subclassification system was less clear for other types of appendiceal cancer outside of mucinous appendiceal adenocarcinomas. For example, in appendiceal goblet cell carcinoma, 59% of analyzed samples were triple-negative for the mutations mentioned [34]. 

**Figure 2:**
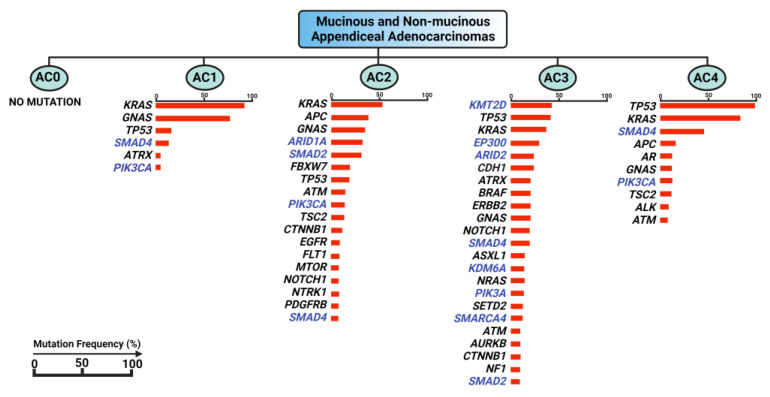
Machine-learning- and genomics-based clustering of appendiceal mucinous adenocarcinoma and adenocarcinoma samples as described by [35], with subtype-defining mutations listed, including mutational frequencies, and mutations in epigenetic-related genes marked in blue. AC0-4: appendiceal cancer subtype 0–4 (nomenclature adopted from Ref. [35] for this figure). Permission to reproduce granted by Springer Nature (license number 5593061501581). Figure created with biorender.com, accessed on 19 July 2023.

Another genomics-based approach to appendiceal cancer classification was attempted by Garland-Kledzik et al. The study used systematic machine learning to explore pre-existing genomic datasets of appendiceal mucinous adenocarcinoma and adenocarcinoma samples using a computer algorithm to create new groups based on transcriptomic similarities evaluating for mutations in a pre-defined set of 41 genes, resulting in five separate subtypes, which were named AC0 to AC4 (Figure 2) [35]. Subtype AC1 showed mutations involved in mucin production and the regulation of organismal growth and endocrine processes, similar to those seen in intraductal papillary mucinous neoplasms (IPMN). The mutational profile of subtype AC2 was similar to that of CRC tumorigenesis and enriched for alterations of apoptotic and autophagy processes. Subtype AC3 exhibited a mutational profile that predominantly affected the epigenomic reprogramming processes, involving genes in histone modification, the regulation of chromatin structure, and DNA binding. Subtype AC4 was enriched in mutations involving differentiation and cell fate pathways, with a genetic profile associated with aggressive adenocarcinoma of the colon and pancreas. Interestingly, subtype AC0 was found to have no mutations or copy number alterations in the evaluated set of genes. Although this warrants more exploration, these unique characteristics may allude to the importance of epigenomic factors and post-transcriptional and post-translational modifications in appendiceal tumor profiling [35].

Overall, the above-mentioned study by Garland-Kedzik et al. found several genetic mutations that may be involved in appendiceal cancer tumorigenesis, including many genes tied to epigenetic signaling pathways and major epigenetic regulators, such as *KMT2D*, *ARID2*, *EZH2*, *SMAD4*, *KDM6A*, *SMARCA4*, *SMAD2,* and *AKT* [35]. Most of these mutations were also identified in other studies; for example, mutations in *KDM6A*, *KMT2D*, *SMARCA4,* and *ARID1A* have been previously reported not only in appendiceal adenocarcinomas but also in appendiceal goblet cell carcinomas [31,32]. Similarly, *SMAD4* mutations have been described in appendiceal adenocarcinomas, low-grade appendiceal mucinous neoplasms, mucinous adenocarcinomas, appendiceal goblet cell adenocarcinomas, and signet ring cell adenocarcinomas [32,36,37]. Although there is no comparative study applying this genomics and epigenetics classification approach to all epithelial appendiceal cancer subtypes, it appears that there may be a significant overlap between the epigenetic pathways dysregulated in appendiceal mucinous adenocarcinomas and adenocarcinomas, appendiceal goblet cell adenocarcinomas, and signet ring cell adenocarcinomas. 

Mutations in major epigenomic regulators could be key factors in epithelial appendiceal malignancies. Consequently, the impact of dysregulated epigenomic pathways and subsequent genetic instability merits further investigation regarding its clinical applicability in diagnostics and prognostication of disease progression and survival in appendiceal neoplasms [31]. Unlike colorectal cancers (CRC), which have a recognized molecular classification known as the consensus molecular subtypes (CMS), a potential molecular classification of AC requires further investigation. Follow-up studies regarding the predictive utility or clinical applicability of molecular classification systems for AC have yet to be published. 

## 3. Genomic Landscape of Appendiceal Cancers vs. Colorectal Cancer

Exploration of the molecular profile of AC primarily leans on genomic studies on colorectal cancers. Mapping the AC genomic landscape has revealed fascinating insights into its molecular profile, but its biological and clinical significance is yet to be fully understood. Importantly, NGS genomic profiling and circulating cell-free DNA (cfDNA) have led to the discovery of variations in genetic alterations among different subtypes of AC, with some sharing similar characteristics of pancreatic and colorectal cancers (CRC) [21]. Nevertheless, data allowing for complete molecular profiling and detection of distinctive features original to AC are still lacking [36]. 

Despite the limitations detailed above, it has become apparent that appendiceal cancers have a unique molecular profile, and their molecular characteristics differentiate AC from other gastrointestinal cancers [17,18,19,20,21]. Even the appendiceal adenocarcinoma, widely considered closest to colorectal cancer among the appendiceal cancer subtypes, demonstrates a distinct molecular profile [38]. Mutational analysis of appendiceal cancers compared to colorectal cancers demonstrated a higher incidence of *GNAS* mutations in appendiceal cancer and a lower incidence of mutations in *APC*, *PIK3CA*, and *TP53*, among others [36]. Of course, it must be noted that there is no comprehensive analysis of mutational frequencies across all appendiceal cancer subtypes available to date.

Although alterations in *KRAS* (62%), *TP53* (36%), *GNAS* (28%), and *APC* (15%) genes occur quite frequently in AC as a whole and have a significantly different mutational pattern from colorectal cancer, no clear associations with histology, grade, or survival have been identified [18]. For example, when stratifying survival based on *GNAS* vs. *TP53* mutations, outcomes were similar to a histopathological stratification. Moreover, while mutational frequencies of these genes were detected to be different between appendiceal tumor subtypes and other GI tumors, the only significant predictors of overall survival were age, grading, and *TP53* mutational status by Cox proportional hazard analysis [17]. In appendiceal adenocarcinomas, only histopathological grade was significant in overall survival, while the mutational status of neither *TP53* nor *GNAS* was significant [36]. Solely in mucinous appendiceal adenocarcinoma, *RAS* vs. *GNAS* vs. *TP53* mutation was predictive of survival [34]. However, within the subtypes of appendiceal mucinous neoplasms, no clear association was observed between mutational status regarding the above-mentioned somatic mutations and overall survival. [37]. However, one study showed that low-grade mucinous neoplasms were associated with concurrent KRAS proto-oncogene, GTPase (KRAS), and GNAS complex locus (GNAS) mutations. In contrast, high-grade mucinous neoplasms were characterized by concurrent *KRAS* and *TP53* mutations, with lower rates of *GNAS* mutations [39]. 

In summary, current data comparing the genetic landscape of appendiceal cancer with colorectal cancer have shown differences between the two cancer types that can potentially aid in distinguishing both cancers and may also help in the classification of appendiceal cancer subtypes. This may contribute to a different perspective on disease staging from the classic histopathological and anatomical evidence. However, no clear added value has been found regarding their correlation with therapeutic strategies or the prognosis of treatment response beyond what has already been demonstrated by histopathological criteria [17,37]. As compared to the other gastrointestinal neoplasms, data on the molecular profiling of appendiceal cancer through epigenomics, proteomics, and metabolomics are lacking. Taken together, these genetic markers alone cannot explain the distinct pathophysiology of appendiceal neoplasms satisfyingly and hence lack clinical applicability. Although the clinical significance of genomic profiling of appendiceal cancer remains undetermined, there is a need to explore additional molecular aspects of appendiceal cancer to understand the pathophysiology of appendiceal cancer to improve early detection and therapeutic outcomes through the identification of potentially actionable targets. 

## 4. The Epigenetic Landscape of Appendiceal Cancers

Epigenetics, or epigenomics, refer to the mechanisms of modification of gene expression that do not result in or require changes to the underlying DNA sequences. These epigenetic modifications are subject to environmental forces and are typically dynamic and reversible. However, they can also be heritable and persist over several generations [40]. The major epigenetic mechanisms include methylation, leading to the suppression or silencing of gene activation, and acetylation, causing the activation of transcription, both of which can take place on histones, affecting large areas of the genome, or in a more specific manner on DNA itself at the CpG sites of the promoter regions of specific genes. The other main categories of epigenetic mechanisms include chromatin remodeling by nucleosome positioning and regulation via non-coding RNAs [41].

Epigenetic changes in malignancy have attracted much attention, especially in gastrointestinal neoplasms, since they often occur early in carcinogenesis and involve key cancer-associated pathways [42,43]. Burgeoning evidence has shown that epigenetic signatures constitute crucial hallmarks of disease pathogenesis. This field has become an area of intensive research for biomarker development and novel therapeutic strategies in the era of precision medicine [43]. The promise of epigenetic markers in early detection has been shown previously by this group in other gastrointestinal malignancies, such as *ADAMTS/BNC1/LRFN/PXDN* in pancreatic cancer, *NDRG4* and *BMP3* in colorectal cancer (as part of Cologuard test), and other DNA methylation markers utilized in the risk stratification of intraductal papillary mucinous neoplasms (IPMNs) [44,45,46,47,48]. However, epigenetics have not yet been well-studied in appendiceal cancers compared to other gastrointestinal neoplasms. 

**Figure 3:**
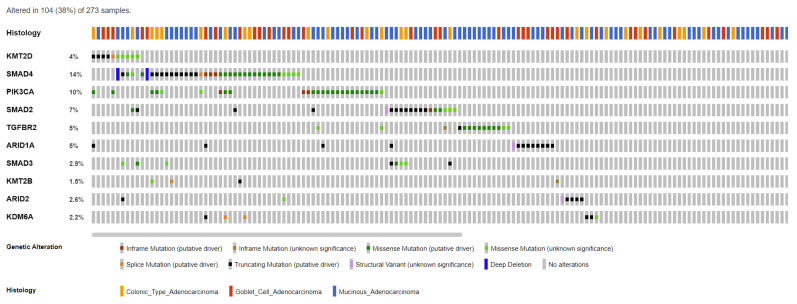
Epigenetic regulatory gene mutations across appendiceal cancer subtypes extracted from the MSK-IMPACT platform created via cBioportal [49,50]. Appendiceal cancer subtypes: mucinous adenocarcinoma (N = 164), goblet cell adenocarcinoma (N = 72), and colonic-type adenocarcinoma (N = 37).

**Figure 4:**
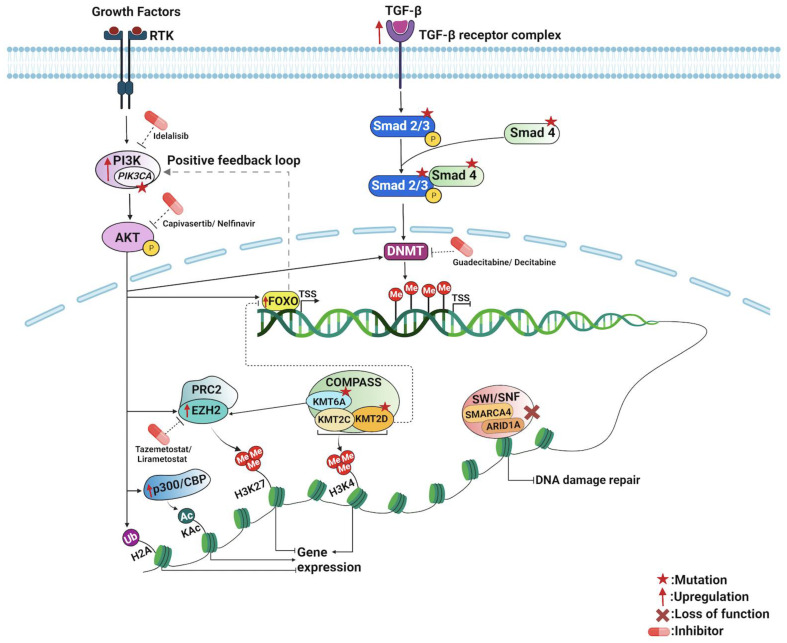
Major epigenetic pathways contributing to oncogenesis in appendiceal cancers and potential therapy-related targets. Ac: acetylation, DNMT: DNA methyltransferase, KAc: lysine acetylation, Me: methylation, P: phosphorylation, RTK: receptor tyrosine kinase, TSS: transcription start site, Ub: ubiquitination. Figure created with biorender.com, accessed on 19 July 2023.

To our knowledge, appendiceal cancers have no established epigenetic alterations or signatures. However, mutational genomic data and pathway enrichment analysis from several molecular studies of appendiceal cancers have revealed genes and pathways that could potentially be involved in epigenetic regulation. Genes such as *PI3KCA*, *SMAD2*, *SMAD3*, *SMAD4*, *KDM6A*, *KTM2C*, *KTM2D*, *ARID1A*, *ARID2*, and *TGFβR2* are found to be commonly mutated in AC (Figure 3, Table 2) [34,36,49,50]. These genes and their pathways are involved in several major epigenetic regulatory mechanisms that may play a key role in appendiceal cancers. As discussed in the following sections, exploring these genes and their regulatory pathways could provide deeper insights into the epigenetic landscape of appendiceal cancers (Figure 4).

## 5. Epigenetic Mechanisms of PI3K/AKT Pathway in Appendiceal Cancers

The Phosphatidylinositol 3-kinase/Protein Kinase B (PI3K/AKT) pathway, known to promote transcriptional competence by priming chromatin structure for subsequent transcriptional activity, is enriched in several appendiceal cancer subtypes across several studies, including appendiceal adenocarcinoma, appendiceal mucinous neoplasms, and appendiceal goblet cell adenocarcinomas [34,36,51,52]. Of note, *PIK3CA*, which encodes for an oncogenic subunit of PI3K, was shown to be one of the top five mutations detected in appendiceal adenocarcinoma (AA) [34,36,53]. A recent study found that *PIK3CA* mutations were found in 10% of AA, most often in the form of in-frame and missense mutations [34,49,50]. Aberrations in or enrichment of the PI3K signaling pathway and its effector gene AKT were more common in high-grade appendiceal mucinous neoplasms. PI3K signaling aberrations are also seen in other gastrointestinal mucinous cancers, suggesting a specific impact on mucinous tumor pathophysiology [36]. 

Although the PI3K/AKT pathway is shown to be enriched in AC, the exact mechanism of how it influences epigenetic changes in AC is still unclear. This may occur via several postulated mechanisms, including global DNA hypomethylation of the genome through DNA methyltransferase (DNMT), as well as upregulation of zeste homolog 2 (EZH2) methyltransferase, which in turn mediates trimethylating promoter-associated Histone H3 Lys27 (H3K27me3) activity. *EZH2* encodes for an enzyme that, as part of the polycomb repressive complex 2 (PRC2), methylates H3K27me3. Thus, PI3K/AKT mediates the activity of a major player in transcriptional repression and epigenetic silencing [52,54]. Interestingly, *EZH2* itself is amplified in a copy number variation analysis of appendiceal mucinous adenocarcinoma and adenocarcinoma samples as well [35]. 

AKT can also stimulate the p300/CREB-binding protein (CBP) coactivator family, which is composed of two closely related transcriptional co-activating proteins, E1A binding protein p300 and the Cyclic adenosine monophosphate Response Element Binding Protein-Binding Protein, or CBP. The p300/CBP complex is responsible for the acetylation of over 100 histone and non-histone substrates, thereby enhancing transcriptional activation [55]. Transcriptional dysregulation, potentially secondary to overactivation of p300/CBP through AKT phosphorylation, was among the top enriched pathways in appendiceal goblet cell adenocarcinomas as compared to other intestinal cancers, which indicates the significance of deregulated epigenetic modulation in the tumorigenesis of appendiceal cancers [51]. Furthermore, AKT-mediated phosphorylation of the histone demethylase KDM5A promotes its cytoplasmic localization, thereby increasing transcriptional competence via H3K4me3 [56]. 

In summary, hyperactivation of the PI3K/AKT pathway has far-reaching consequences leading to transcriptional dysregulation through several pathways, many of which appear to act in a tumor-promoting manner.

## 6. Epigenetic Mechanisms of TGF-β/SMAD Pathway in Appendiceal Cancers

Disruptions in transforming growth factor beta (TGFβ) signaling can affect epigenetic gene silencing, particularly in relation to the epithelial–mesenchymal transition (EMT). This process is mediated through activation of the Suppressor of Mothers Against Decapentaplegic (SMAD) complexes, specifically SMAD2/3, which associate with SMAD4 for nuclear translocation, where they induce transcription of EMT-related transcription factors, such as Snail Family Transcriptional Repressor 1 and 2 (SNAIL and SLUG), Zinc Finger E-box Binding Homeobox 1 and 2 (ZEB1 and ZEB2), Twist-related Protein 1 (TWIST1), and others [57]. *SMAD2/3* and *SMAD4* are mutated across multiple appendiceal cancer subtypes and in malignancies of other tissues [35,36,37]. Cancer cells undergoing the EMT process through overactivation of TGFβ signaling exhibit sustained hypermethylation of promoters and subsequent loss of expression in downstream cell-junction-related effector genes, including cadherin 1 (*CDH1)* and claudin 6 (*CLDN6)*. 

TGFβ stimulation in ovarian cancer cells treated with non-specific DNMT inhibitor SGI-110 or guadecitabine led to increased activity and nuclear localization of particularly DNMT1 and prevention of *CDH1* silencing, which suggests a TGFβ-DNMT1-CDH1 pathway. Similarly, the downregulation of *CLDN6* expression is mediated via a TGFβ-activated SMAD2/DNMT1 axis. This demonstrates a direct link between TGFβ signaling and epigenetic regulation mechanisms, such as DNA methylation, specifically mediated via SMAD2 and DNMT1 [58,59]. Similarly, epigenetic silencing of *RunX1T1* through loss-of-function mutation of *SMAD4* in the setting of aberrant TGFβ signaling has been linked to promoting tumorigenesis in ovarian cancer. Interestingly, this effect in ovarian cancer appears mediated via DNA methylation and precedes histone modifications [60].

## 7. Chromatin Remodeling and Transcription in Appendiceal Cancers

### 7.1. SWI/SNF Chromatin Remodeling Complex 

One of the essential epigenetic modulators is SWItch/Sucrose Non-Fermentable (SWI/SNF), one of four major families of chromatin-remodeling complexes and a key regulator of nucleosome positioning and modifier of gene enhancer accessibility. SWI/SNF has been shown to mediate cell differentiation and was also discovered to play a role in DNA damage repair by modifying chromatin structures around the site of DNA damage and recruiting proteins belonging to the DNA damage repair machinery [61,62]. The SWI/SNF complex consists of multiple subunits, several of which have been indicated to possess oncogenic potential [61,63]. Two subunits, ATPase SMARCA4 and complex-associated factor ARID1A, have been reported to be involved in DNA damage repair by assisting in homologous recombination-mediated DNA repair and non-homologous end joining at sites of double-strand breakage [64,65]. Both *SMARCA4* and *ARID1A* are mutated in appendiceal goblet cell carcinoids, mixed goblet cell carcinoid–adenocarcinomas, and some appendiceal mucinous adenocarcinomas and adenocarcinomas [31,35]. These mutations, and most other mutations affecting genes encoding for the SWI/SNF complex, lead to a loss of function of the respective proteins and have been linked to tumor progression in several malignancies, marking these genes as tumor suppressors [61].

### 7.2. COMPASS Chromatin Remodeling Complex 

Another significant chromatin-remodeling complex is Complex Proteins Associated with Set1 (COMPASS). One of its main catalytically active components is the lysine-specific demethylase 6A histone demethylase KDM6A (or UTX) [66,67]. The type 2 lysine methyltransferases C and D (KMT2C and KMT2D) are enzymatically active by methylating the histone 3 lysine 4 (H3K4me3). Their involvement in the regulation of gene expression is widespread. Mutations in *KMT2C*, *KDMT2D*, and *KDM6A* have been linked to the development of congenital disorders, emphasizing their importance for mammalian cell function through all stages of development and across various tissue types [68]. KMT2D has been studied extensively in prostate cancer and has also been shown to activate the PI3K/AKT pathway and support epithelial–mesenchymal transition pathways in carcinogenesis [69]. *KDM6A* and *KMT2D* mutations have been reported in the appendiceal goblet cell carcinoid, mixed goblet cell carcinoid–adenocarcinoma, and some appendiceal mucinous adenocarcinomas and adenocarcinomas [31,35]. 

Interestingly, the effects of *KDM6A* mutation are not uniform across different cancers and likely depend on the transcription factors it interacts with in each specific tissue type. For example, KDM6A has been implicated as a tumor suppressor in gastrointestinal malignancies. However, it appears to influence oncogenic transcription factors’ activity in hormonally driven cancers. *KDM6A* has also been linked to EZH2; loss-of-function mutations in *KDM6A* seem to affect transcriptional repression by EZH2 and have been shown to increase susceptibility to treatment with EZH2 inhibitors [67,70]. 

### 7.3. The Forkhead Box O (FoxO) Transcription Factors 

The Forkhead box O (FoxO) family of transcription factors regulates the expression of genes in crucial cell physiological events, including apoptosis, cell cycle control, glucose metabolism, and oxidative stress resistance. A central regulatory mechanism of FoxO proteins is phosphorylation by AKT downstream of PI3K, which leads to the disruption of FoxO DNA binding [71,72,73,74]. In addition, an association has been found between FoxO3 and the COMPASS-associated methyltransferase KMT2D, as loss of KMT2D function was found to cause enhanced vulnerability to DNA damage through the suppression of antioxidative gene transcription caused by diminished DNA binding of FoxO3, likely in a PI3K/AKT-independent manner [69]. FoxO signaling is enriched in appendiceal goblet cell adenocarcinoma compared to colorectal adenocarcinoma [51].

## 8. Exploration of Potential Epigenetics-Based Biomarkers for Novel Therapeutic Targets

The discovery of gene mutations in the epigenetic field has unraveled exciting new areas of investigation with great potential regarding developing novel targeted therapies, which could then be applied to treating appendiceal neoplasms along with other malignancies. Some of these concepts have already been investigated as prospective drug targets in various cancers. For example, promising new data demonstrate that tumors with *KDM6A* mutations, leading to functional deficiency of the encoded protein, respond particularly well to mTOR inhibitors, such as everolimus or sirolimus, which are FDA-approved for the treatment of renal cell carcinoma, among others, because of the ensuing loss of epigenetic transcriptional regulation resulting in hyperactivation of mTORC1 [67]. Additionally, idelalisib, a direct PI3K inhibitor, was recently approved for treating relapsed chronic lymphocytic leukemia (CLL). Ongoing studies focus on developing and testing new AKT inhibitors to counteract its hyperactivation, which ultimately promotes hypermethylated DNA states linked to carcinogenesis [52]. A recently published phase 3 trial on the AKT inhibitor capivasertib shows promising data on the efficacy of AKT inhibition in prolonging progression-free survival in relapsed hormone-receptor breast cancer patients [75]. Another AKT-inhibiting compound currently under later-stage clinical investigation is nelfinavir, a human immunodeficiency virus (HIV) protease inhibitor, which has been shown to reduce AKT phosphorylation and downregulate the PI3K/AKT pathway [76].

Interestingly, FoxOs have also been able to re-activate the PI3K/AKT pathway as part of a feedback loop mechanism, which is exploited in certain malignancies to build resistance to PI3K/AKT inhibitors [74,77]. This stresses the value of FoxO as a potential biomarker when considering PI3K/AKT inhibitor-based treatment, especially in tumors that have been detected to possess both PI3K/AKT and FoxO enrichment, like appendiceal goblet cell adenocarcinoma subtype [51]. 

Exciting advances have also been made in recent years in EZH2-targeted therapies. As detailed above, *EZH2* is mutated in specific forms of appendiceal cancers, and several of the other epigenetic regulators found to be mutated in appendiceal neoplasms have been linked in some form to *EZH2* overexpression or hyperactivation as well, most prominently PI3K/AKT, as well as KDM6A and specific subunits of the SWI/SNF complex. This makes EZH2 a prime therapeutic target, and several compounds have been developed since EZH2 inhibitor Tazemetostat was FDA-approved for advanced epithelioid sarcoma as well as relapsed or refractory follicular lymphoma, with several ongoing phase 1 and 2 clinical trials investigating similar drugs, such as SHR2554 and CPI-1205 (or lirametostat) in small intestine neuroendocrine tumors and relapsed or refractory B-cell/T-cell and Hodgkin’s lymphomas, respectively [78,79,80]. Another study linked EZH2-mediated epigenetic changes to chromatin density to increased resistance to DNA damage in cells with concurrent p53 mutation and presented data suggesting that resistance to treatment approaches with chemotherapy and radiation as conferred by *p53* mutation could be overcome, at least in part, by EZH2 inhibition [81,82]. However, the direct targeting of these mutations or their affected pathways is not the only attainable treatment approach. There are, for example, encouraging data proposing the utility of existing DNA damage repair inhibitors in tumors with *KMT2D* mutations. These findings align with the increased susceptibility to DNA damage found in KMT2D-deficient tumors, as evidenced by increased sensitivity to PARP inhibitors [69]. Similar findings were obtained in tumors with mutations affecting the SWI/SNF complex. Specifically, PARP inhibitors are under investigation in several trials for tumors with *ARID1A* mutation, based on the involvement of the SWI/SNF complex in DNA damage repair and therapeutic vulnerability observed in preclinical studies [61]. 

Another potential avenue of treatment options targeting epigenetic players is plant homeodomain (PHD) co-fingers, found, for example, in KDM5A, a reader/effector protein activated by the PI3K/AKT pathway and acting on H3K4me3, as previously detailed above [52]. There is some pre-clinical evidence based on small in vitro studies suggesting that KDM5A, via PHD3, could be inhibited by already-FDA-approved agents, including amiodarone derivatives and disulfiram, as well as novel small molecule cyclopeptides [83,84,85]. Further studies are necessary, however, to prove the clinical efficacy, safety regarding therapeutic windows, and adverse effects of this new indication, and the feasibility of the administration of these agents as PHD finger inhibitors and epigenetic modulators.

A tremendous breakthrough in medical oncology was achieved with the introduction of immune checkpoint inhibitors. Exciting data propose the potential for treatment synergism between immunotherapy and epigenetic drugs, such as DNA demethylating agents. It has been shown that treatment with this class of drugs creates an Interferon-mediated immune response within the tumor microenvironment of hematological, ovarian, and colorectal cancers [86,87,88]. This is thought to enhance the efficiency of the antitumoral immune response, which has been hypothesized to increase even further in combination with immune checkpoint blockers. Furthermore, dysregulation of epigenetic silencing by DNMT1 inhibition via PI3K/AKT hyperactivation and aberrant activation of the TGFβ signaling pathway have been unmasked as key drivers behind immune evasion and lack of response to immunotherapy [89]. Other studies have revealed enhanced sensitivity to immune checkpoint blockade in tumors carrying SWI/SNF complex mutations. *ARID1A* deficiency led to significantly reduced tumor burden and prolonged survival upon immunotherapy compared to wild-type tumors in studies of ovarian and gastric cancers [61]. Likewise, deviant transcriptional regulation due to inhibition of EZH2 has been implicated in correlating with the immunogenicity of tumor cells and immune silencing in the tumor microenvironment. The utility of combination therapies with EZH2 inhibitors and immune checkpoint blockers remains to be investigated further, with several clinical trials underway [81].

Further studies are needed to evaluate possible biomarkers regarding their predictive power and gene mutations regarding their clinical applicability for treatment-related decision making. However, the above-mentioned studies and clinical trials show very promising results and we see great potential for the recruitment of new epigenetic-related therapeutic avenues in relapsed or otherwise treatment-refractory cases of appendiceal cancer, specifically for the subtypes of appendiceal mucinous adenocarcinomas and adenocarcinomas, appendiceal goblet cell and signet ring cell adenocarcinomas which exhibit favorable, epigenetics-related mutational profiles.

## 9. Epigenetics-Based Biomarkers for Monitoring, Surveillance, and Prognostication of Appendiceal Cancers

Epigenetic regulators present promising opportunities for developing biomarkers and translating treatment strategies from other malignancies into appendiceal cancers. However, the question remains whether they can provide additional information for prognostication of response to different therapies and, most importantly, survival. More data are needed to comment on this question, especially in appendiceal neoplasms. 

It has been described that mutations affecting certain epigenetic-related pathways could be linked to unfavorable outcomes. Unsupervised hierarchical clustering analysis of appendiceal tumor expression profiles showed that AKT pathway activation and upregulation of pathways involved in epithelial–mesenchymal transition, like TGFβ/SMAD, were associated with a decrease in both overall and progression-free survival [19]. Additional studies corroborate the possibility of prognostic validity of TGFβ pathway hyperactivation. For example, a panel of gene expression changes, including *TGFβ* upregulation, was identified to stratify peritoneally metastasized appendiceal tumor specimens, all of which were determined to be low-grade by histopathology, with two groups showing a significant difference in overall survival [90].

Additional findings link *KMT2D* deletion in prostate cancer cells to increased sensitivity to both conventional chemotherapy and PARP inhibitors, making *KMT2D* expression status a potential novel biomarker for the prognosis of the treatment response [69]. Furthermore, specific epigenetic biomarkers have been hypothesized to predict to which degree a tumor might respond to immunotherapy. This theory is based on existing knowledge of how immune-related biomarkers which are currently in use, such as the expression of *PD-L1*, tumor-associated antigens, or HLA, are subject to epigenetic regulation and may undergo extensive epigenetically driven alterations at various stages throughout the disease process and even during treatment [89]. Unfortunately, data applying these findings to appendiceal cancers are still lacking. However, existing studies are highly encouraging and inspire further investigation.

Many genes involved in the epigenetic modulation discussed above can potentially become biomarkers in diagnosing and managing appendiceal cancers. They may aid in classifying subtypes, therapeutic targets, prognosis, monitoring, and surveillance. However, there are additional considerations to take into account in the development of robust biomarkers. Not only do targets need to be identified, but they must also be validated in large-scale studies. Current data are based on smaller, often retrospective sequencing analyses with limited sample sizes. Further investigation is necessary to build on these data and dissect the epigenetic landscape of appendiceal oncogenesis. 

In addition, clinically feasible, safe, and cost-effective testing needs to be made available [91]. Liquid biopsy, a blood-based analysis of circulating tumor DNA (ctDNA), has emerged as a prominent noninvasive procedure with minimal risk of complications compared to conventional tissue biopsies [92]. Circulating tumor DNA (ctDNA) is a part of cell-free DNA (cfDNA). Cell-free DNA consists of DNA fragments that are released into the blood plasma as part of a physiological process upon apoptosis or lysis of cells. The DNA fragments stemming from tumor cells in the body are called ctDNA and have the potential to carry the entire genetic information of the tumor [93,94]. Liquid biopsies allow for highly personalized tumor analysis via next-generation sequencing. Studies investigating the feasibility of liquid biopsies in appendiceal cancers have shown that the analysis of ctDNA is a comparable alternative to tumor tissue biopsy, which can be technically challenging for the physician and associated with a higher risk of procedure-related morbidity for the patient. However, this is currently not yet available as a standard diagnostic [92,95]. 

Further studies are needed to confirm the safety and feasibility of testing for each specific novel epigenetic biomarker under investigation. Nonetheless, whole-genome analysis of changes in methylation patterns may yield a wealth of information for clinicians dealing with appendiceal cancers, primarily as part of an individualized oncology approach, and even more so if utilized in liquid biopsy format. 

## 10. Conclusions

The intricacies of epigenetic alterations and mechanisms in appendiceal neoplasms are still largely unknown. However, several epigenetic mechanisms have been postulated based on currently available data, which hold highly promising potential for clinical applicability regarding novel diagnostics and prognostication in appendiceal cancers. Further studies are necessary to validate previous findings in a methodical, epigenomics-centered, and translational approach. Epigenetics-based biomarkers may be the key to a deeper understanding of epithelial appendiceal cancer pathophysiology and aid in uncovering actionable targets for disease monitoring in appendiceal cancers. Ultimately this could enable clinicians to prognosticate responses to various therapy approaches, estimate the risk of progression or relapse, and predict overall survival in their patients, thereby making personalized oncology a reality in managing and treating appendiceal neoplasms.

## Figures and Tables

**Table 1:** Mutational frequencies of select genes comparing GCA, ANET, and AA as described in [32]. Goblet cell adenocarcinoma (GCA); appendiceal neuroendocrine tumor (ANET); appendiceal adenocarcinoma (AA).

Goblet Cell Adenocarcinoma (GCA) N = 53		Appendiceal Neuroendocrine Tumor (ANET) N = 14		Appendiceal Adenocarcinoma (AA) N = 428	
Genes	Percent Mutation (%)	Genes	Percent Mutation (%)	Genes	Percent Mutation (%)
*TP53*	24	*KRAS*	28.6	*KRAS*	60.4
*ARID1A*	15.4	*APC*	28.6	*TP53*	37.0
*SMAD4*	9.4	*TP53*	14.3	*GNAS*	34.4
*KRAS*	7.5	*CDH1*	7.7	*ARID1A*	20.0
*BRAF*	3.8	*BRAF*	7.7	*SMAD4*	18.3
*FBXW7*	3.8	*BCOR*	7.7	*APC*	11.7
*CDH1*	3.8	*BRCA2*	7.1	*PI3KCA*	7.0
*KDM6A*	2.7	*FANCA*	7.1	*RNF43*	5.9
*APC*	1.9	*ERBB2*	7.1	*ATM*	5.0
*PIK3CA*	1.9	-	-	*BRAF*	4.0
*ATM*	1.9	-	-	*FBXW7*	3.6

**Table 2:** Percentage of epigenetic regulatory gene mutations in mucinous adenocarcinomas, non-mucinous adenocarcinomas, and goblet cell appendiceal cancer subtypes extracted from MSK-IMPACT platform created via cBioportal [49,50].

Genes	Percentage of Gene Mutations (%)		
	Mucinous Adenocarcinoma N = 164	Goblet Cell Adenocarcinoma N = 72	Appendiceal Adenocarcinoma (Non-Mucinous) N = 37
*KMT2D*	3.0	4.2	5.4
*KMT2B*	0.9	4.5	5.9
*KDM6A*	0.6	2.8	8.1
*SMAD2*	6.7	4.2	10.8
*SMAD3*	3.0	-	8.1
*SMAD4*	11.0	12.5	21.6
*PIK3CA*	7.3	5.6	27.0
*TGFBR2*	7.3	1.4	2.7
*ARID1A*	3.0	8.3	2.7
*ARID2*	1.8	2.8	2.7

## Data Availability

Not applicable.
